# User-Centered Design and Development of the Modular TWIN Lower Limb Exoskeleton

**DOI:** 10.3389/fnbot.2021.709731

**Published:** 2021-10-07

**Authors:** Matteo Laffranchi, Stefano D'Angella, Christian Vassallo, Chiara Piezzo, Michele Canepa, Samuele De Giuseppe, Mirco Di Salvo, Antonio Succi, Samuele Cappa, Giulio Cerruti, Silvia Scarpetta, Lorenzo Cavallaro, Nicolò Boccardo, Marialaura D'Angelo, Claudia Marchese, Jody A. Saglia, Eleonora Guanziroli, Giacinto Barresi, Marianna Semprini, Simone Traverso, Stefano Maludrottu, Franco Molteni, Rinaldo Sacchetti, Emanuele Gruppioni, Lorenzo De Michieli

**Affiliations:** ^1^Rehab Technologies Lab, Istituto Italiano di Tecnologia, Genova, Italy; ^2^Centro Protesi INAIL, Istituto Italiano per l'Assicurazione contro gli Infortuni sul Lavoro, Vigorso di Budrio, Italy; ^3^Villa Beretta Rehabilitation Centre, Valduce Hospital, Costa Masnaga, Italy

**Keywords:** exoskeleton, mechatronics, healthcare robotics, user-centered design, wearable robotics

## Abstract

For decades, powered exoskeletons have been considered for possible employment in rehabilitation and personal use. Yet, these devices are still far from addressing the needs of users. Here, we introduce TWIN, a novel modular lower limb exoskeleton for personal use of spinal-cord injury (SCI) subjects. This system was designed according to a set of user requirements (lightweight and autonomous portability, quick and autonomous donning and setup, stability when standing/walking, cost effectiveness, long battery life, comfort, safety) which emerged during participatory investigations that organically involved patients, engineers, designers, physiatrists, and physical therapists from two major rehabilitation centers in Italy. As a result of this user-centered process, TWIN's design is based on a variety of small mechatronic modules which are meant to be easily assembled and donned on or off by the user in full autonomy. This paper presents the development of TWIN, an exoskeleton for personal use of SCI users, and the application of user-centered design methods that are typically adopted in medical device industry, for its development. We can state that this approach revealed to be extremely effective and insightful to direct and continuously adapt design goals and activities toward the addressment of user needs, which led to the development of an exoskeleton with modular mechatronics and novel lateral quick release systems. Additionally, this work includes the preliminary assessment of this exoskeleton, which involved healthy volunteers and a complete SCI patient. Tests validated the mechatronics of TWIN and emphasized its high potential in terms of system usability for its intended use. These tests followed procedures defined in existing standards in usability engineering and were part of the formative evaluation of TWIN as a premise to the summative evaluation of its usability as medical device.

## Introduction

Spinal cord injury (SCI) is a particularly critical condition which often leads to permanent disability, use of wheelchair, and several secondary clinical complications. These complications inevitably impact on physical, mental, social and economic conditions of SCI patients (WHO, 2013). As a consequence, traditional therapy based on manually assisted mobilization of the patients has been introduced to prevent, or even cure, many complications. Nevertheless, this approach presents a series of difficulties: the therapist has often to perform the treatment in awkward positions, experiencing early fatigue, which may result in poor therapeutic outcome (Foulds et al., 2014). Furthermore, traditional assisted gait retraining of incomplete subjects is often critical, given the large number of joints to be simultaneously managed and to the consequent poor repeatability and reproducibility (Foulds et al., 2014).

In this scenario, exoskeletons are a valid tool that can easily overcome the mentioned limitations: they can intensify the training, allow the patient to autonomously walk over ground, for longer duration, and reproduce rhythmically correct movement patterns. Motivated by this, researchers have been developing a vast range of robotic exoskeletons for SCI patients. The current leading products are Rewalk (Esquenazi et al., 2012), Ekso (Milia et al., 2016), and Indego (Farris et al., 2014), which have demonstrated their effectiveness in the prevention of secondary complications, patient's health and improvement of the quality of life in several clinical studies.

However, these devices are typically adopted in the clinical context. Others, such as the MINDWALKER (Wang et al., 2015) and the Symbitron (Meijneke et al., 2021), are examples of striking research devices, which are however far from effectively and autonomously being used for independent training. Unfortunately, as the health benefits resulting from exercising largely depend on its frequency of execution, duration, and continuity (Foulds et al., 2014), SCI patients should be able to autonomously use the exoskeleton as a personal device to keep the training frequency high and hence fully benefit from the efficacy of exoskeleton-based therapy.

Nevertheless, current exoskeletons suffer from poor usability (Lajeunesse et al., 2016), which is the main cause that prevents them from being exploited to solve problems of everyday life. Poor usability is the consequence of the lack of several crucial points which are common in most current devices: they are frequently indicated as difficult to be worn autonomously (Gorgey, 2018), mainly due to the considerable weight, size, and “monolithic” structure, which creates difficulties in donning-doffing, transportation and general device handling (Fritz et al., 2019). Moreover, even among the most prominent commercial exoskeletons, surprisingly only the Parker Indego exoskeleton has made an attempt to solve these issues by improving usability contextualized into personal use. Indeed, it is the only available device that can be disassembled without the need of tools for ease of transportation, and which claims wheelchair compatibility. The latter point is essential as the vast majority of SCI patients use the wheelchair as primary mobility aid (Berkowitz et al., 1998). As high usability is an essential prerequisite to increase the effective adoption of exoskeletons as personal devices in everyday life, their design approach should be completely revised, from traditional technology-centered engineering design processes, to user-centered methods which guarantee the direct addressment of user needs (Masia and Vitiello, 2020).

In this work, we present a novel lower limb exoskeleton named TWIN, for personal utilization of SCI patients, that was developed to address SCI patients' needs directly by means of user-centered design. This process initially involved the final users, which indeed confirmed autonomous usage, that is strictly correlated to usability, as top priority among a set of critical features. This information was then employed for drafting the general architecture layout of the exoskeleton and the requirements of the device to guide its development. Following these procedures (based on medical device design practices), the TWIN exoskeleton was hence conceived to maximize usability, by facilitating donning, transportation, and general device handling. We indeed believe that enabling autonomous usage of these devices can increase the frequency of exoskeleton-based training which is necessary for the successful outcome of the training program (Foulds et al., 2014). Moreover, its structural elements come in different sizes to accommodate the anatomy of the specific patient and are compatible with wheelchair use. Preliminary evaluation was carried out on healthy subjects and an SCI patient to validate the mechatronic viability of the device alongside its ergonomics, considering safety, comfort, and other aspects of usability that must be assessed far before the clinical trials.

## User Needs and Design Requirements

The design process that enabled the user-centered development of TWIN followed two consecutive phases as listed below:

1. An “exploratory phase” was characterized by the investigation of the user needs to establish a set of user-centered design requirements (Martin et al., 2006; Chandran et al., 2020).
2. A “formative phase” which was designed based on the international standard IEC 62366-1:2015 on the application of usability engineering to medical devices (Scherer and Gouveia Filho, 2019). This phase sustained the participatory evaluation processes adopted to progressively improve the system usability, iteration by iteration (Simonsen and Hertzum, 2010).

**Figure 1:**
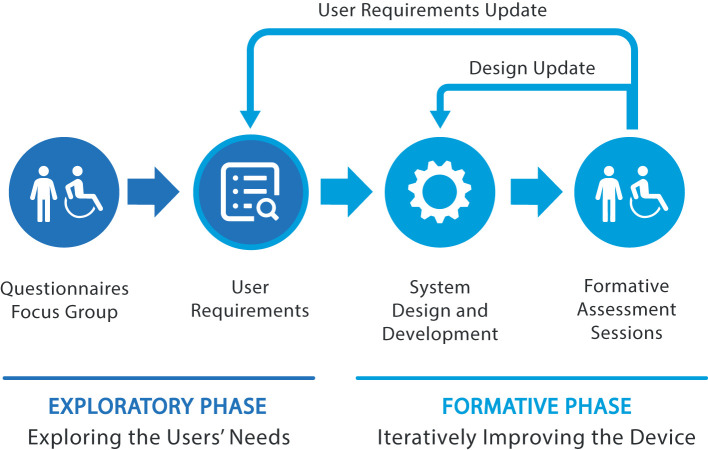
The design process employed in TWIN consisted of two main stages: the exploratory and formative phases, which directly involved users and the stakeholders.

This process can be summarized in the conceptual scheme shown in Figure 1. After completion of the exploratory phase, the formative phase which follows includes iterations where the design of the device and its subsystems are continuously assessed and updated to comply with the set user requirements. The formative phase may also update the design requirements of the device to comply with possible additional request emerged during the above-mentioned participatory evaluation.

Considering the literature in wearable robotics, the most characteristic trait of our approach is the adoption of methodologies typically exploited in industrial contexts which target at achieving the development of systems with high technology readiness level (TRL), and according to users' needs. Indeed, the TWIN exoskeleton was considered a medical device in all its development phases. Consequently, we adopted the perspective of user research and the international standards of usability engineering for medical devices (Privitera et al., 2017; Bitkina et al., 2020). This choice constitutes one of the original contributions provided by this manuscript to the domain of wearable robotics research.

### Exploratory Phase: Analysis of User Needs

As part of the exploratory phase, we first conducted a preliminary series of studies based on questionnaires and focus groups involving patients, aimed at understanding their primary needs and requirements (Shah and Robinson, 2008). This information was then translated into requirements for new device concepts and control strategies that were taken into account for the implementation of the TWIN exoskeleton.

This investigation was conducted in collaboration with the Centro Protesi INAIL (i.e., the Italian National Institute for Insurance against Accidents at Work) in Vigorso di Budrio (Italy) and with the ISTUD foundation. The study comprised a questionnaire and three focus groups that took place in three SCI centers assisting SCI patients. Data collection was performed between June and October 2014 and inclusion criteria were: age >18 years; at least 6 months from spinal unit hospitalization; no related psychopathological comorbidity. Included subjects granted the authorization to treat personal data, in accord with national laws (D.Lvo. 196/2003) and GDPR regulation. The questionnaire was made available electronically on different platforms and web resources that are usually accessed by spinal patients and was advertised in many hospitals and spinal units. Questions regarded: (1) *socio-demographic information*; (2) SCI *information*; (3) *domestic life*; (4) *work/school*; (5) *free time, hobby, and sport*; (6) *health condition and quality of life*; (7) *autonomy in everyday life and transfers* (autonomy in domestic and non-domestic duties, and autonomy with respect to different ways of travelling); (8) *standing* (habits, perception of standing benefits, opinions related to strength, and limits of available orthoses); (9) *exoskeleton* (impressions of available devices, of their utility and utilization, preferences, and interest for a possible new device). We collected 107 questionnaires. The interviewed population was composed by 79% males with average age of 44 years, confirming the results of a previous study on the Italian population (Pagliacci et al., 2003). Table 1 summarizes the type and level of lesions of the interviewed patients.

**Table 1:** Occurrence of lesion based on type and level.

**Type**			**Level**			
**Complete**	**Incomplete**	**Not specified**	**Cervical**	**Dorsal**	**Lumbar**	**Not specified**
69%	27%	4%	22%	68%	6%	2%

We also performed focus groups, in which a moderator stimulated the discussion between a group of selected patients and collected their response, impressions, and feelings toward the covered topics. A co-creation session followed, where subjects were asked to brainstorm and provide potential use case scenarios and design inputs. Focus groups were organized in three Italian spinal cord centers: Montecatone Rehabilitation Institute (Imola, 17 people−14 males); Unità Spinale Unipolare of Careggi hospital (Florence, 7 people−6 males); Unità Spinale Unipolare of Alesini hospital (Rome, 8 male people).

The data analysis that followed focused on the aspects that were significant to the development of the exoskeleton device, to draft conclusions which could in turn be useful for the following design phases. Therefore, we aimed at understanding: (1) main adversities encountered during daily life, (2) opinion and perception of SCI subjects on the benefits obtained by adopting a standing posture, and (3) opinion on the use of exoskeletons.

Regarding adversities encountered in personal life, collected data reported shared difficulties in traveling and commuting, mostly related to architectonic barriers, both during travel and at destination. Indeed, many people complained about the lack of autonomy during commuting, which is often by car and affected by the difficulties of transferring from wheelchair to car and vice versa. Only 58% of the interviewed people found this transfer easy to perform, while others rely on external help. It must also be noted that traveling and visiting friends were indicated as the free time activities mostly affected by the injury.

**Figure 2:**
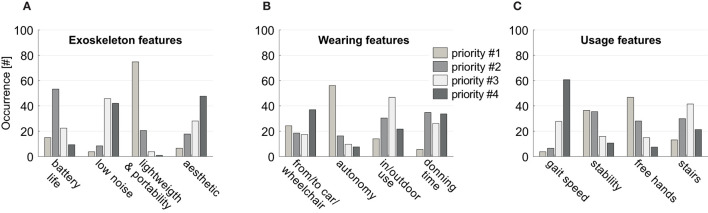
Questionnaire outcomes. Perceived priorities for: **(A)** exoskeleton features, **(B)** exoskeleton wearing, **(C)** exoskeleton use. Histograms represent the number of subjects indicating the features reported in the abscissa as top priority (light gray bars), second priority (gray bars), third priority (white bars), or fourth priority (dark gray bars).

We observed the attitude toward the standing posture and found that 58% of the interviewed subjects used devices for standing that are not orthoses, on average 3 days per week for around 90 min per session. Only 8% used a knee-ankle-foot orthosis (KAFO) and they all judged the device very useful. Around 29% of the participants declared that they did use KAFO for some time but then they abandoned the device for diverse reasons, such as fatigue, time issue, difficulty of use, etc. However, almost the totality of the sample (93%) believed in the beneficial effects of standing on rehabilitation and for improving health condition and thus demonstrated a positive attitude toward the development of a device truly designed around their needs. Addressing issues related to the features of exoskeleton, 92% of people with incomplete lesion and 74% of subjects with complete lesions demonstrated interest in them, and they indicated lightweight and portability as the top priority, followed by battery life (>2 h), low noise, and aesthetics (Figure 2A). With respect to battery life, given that a typical exoskeleton session performed by an expert user lasts about 1 h, we decided to set 2 h as the lower bound, so as to allow the use of the device for 1–2 training sessions.

Regarding device wearing features, the highest priority was represented by the possibility of being worn and removed quickly and autonomously, also from/to the wheelchair or the car, and to be used both indoor and outdoor (Figure 2B). Finally, on usage features, the interviewed sample stated that standing without arm support and stability, are more important than speed or the possibility of access stairs (Figure 2C). Overall, what emerged were specific suggestions for an effective application of robotics to people with SCI. Their feedback could be summarized in two main key needs, i.e., the device should be used autonomously and should be practical to facilitate its employment during its daily usage. Based on the results reported in Figure 2 and on the feedback given during the focus groups, the main user-driven requirements for the development of the TWIN exoskeleton were drafted.

As expected, the first two requirements are directly linked to autonomy and have therefore been prioritized. Stability during use is directly linked to the safety of the device, whereas cost effectiveness, although not reported in Figure 2, has been added because all the subjects verbally complained about the current cost of these machines during focus groups. Finally, from the focus groups, we had insight that long battery life is required to make the subjects feel safe and autonomous during the session. These requirements were then employed to draft conceptual layouts of the machine in cooperation with industrial designers, so as to co-develop the device to address usability as well as technical issues. These aspects were all taken into account for the design of the TWIN exoskeleton. Another priority that is not listed in the requirements was represented by the possibility of reaching a standing posture with free hands, but the practical implementation of this point required solutions that were in contrast with most of the other priorities and was thus left for future developments.

This preliminary study triggered the development of TWIN and its formative assessment.

### Formative Phase: Iterative Improvements of the Device

The international standard IEC 62366-1:2015 and its related technical report IEC 62366-2:2016 (Kendler and Strochlic, 2015) indicate methods to be used for the formative evaluation phase to assess the usability of medical devices. This evaluation must be carried out starting from the most preliminary design iterations throughout to the final prototype so as to guarantee that usability is considered during the whole design process. Accordingly, the role of the formative evaluation is to guide the iterative and participatory design and development of the system for progressively resolving its most critical usability issues. The standard IEC 62366-1:2015 defines how formative evaluation can exploit a heterogenous set of techniques to collect individual feedback, including qualitative observations expressed (spontaneously or partially guided by a user researcher) by a limited number of subjects for checking major usability problems of each preliminary prototype. These subjects can include both final users and stakeholders—e.g., caregivers and orthopedic technicians who, according to their own expertise, heuristically predict critical flaws in systems usability (Bitkina et al., 2020).

The standard IEC 62366-1:2015 also establishes that, after completion of the formative evaluation, the design process will be concluded with a summative assessment, which is defined as the validation of the system on usability and safety aspects before its certification and release. Differently from the formative assessment, the summative assessment typically requires a comprehensive (especially quantitative and objective) test of the ultimate iteration of the system, possibly with the involvement of numerous users. Before proceeding with the summative assessment, clinical trials are currently performed on the device for the advanced stages of the formative phase.

In order to improve the methodological rigor of our formative assessment, its advanced sub-phases will focus on the correlation of subjective and objective indices to obtain consistent measures of exoskeleton usability. First of all, the protocol will include standardized questionnaires on perceived (subjective) usability: the System Usability Scale (Brooke, 1996) to investigate the caregivers' (which will manage the GUI of the TWIN software) perspective; the NASA-TLX (Hart and Staveland, 1988) for deepening our understanding of the patients' cognitive load in using the device. The questionnaire scores of the users will be analyzed according to (objective) motor and physiological data collected during the exoskeleton usage (and exploited to assess the user's effort, for instance) (Kozlowski et al., 2015). We plan to analyse observable patients' behaviors during autonomous donning/doffing and in the execution of training. Such a multimodal approach is just a demonstration of our plans in matching the subjective and the objective measures for showing a final, and comprehensive, estimation of system usability which will be carried out in the near future.

This paper focuses on the first 36 months of exploratory and formative phases of TWIN. Initially, 10 healthy volunteers participated to the tests. They had no neurological or muscular diseases. Once the TWIN device underwent a first series of iterative improvements and was assessed to be ready for user trials, a second stage involved one 31-year-old SCI patient with a complete D5 lesion and experience in the use of lower limb exoskeletons. All the participants gave written and informed consent before their inclusion in the study. The tests respected the standards of the Declaration of Helsinki (rev. 2013) and were formally approved by the ethics evaluation committee Comitato Etico Interaziendale Bologna-Imola of the Pharmaceutical Department U.O.C. Farmacia Ospedale Maggiore, Bologna, Italy (Protocol number: CP-POR1-01 ver.01).

During the sessions of the formative phase, the evaluation was carried out in empty areas, under the supervision of qualified personnel to ensure the safety of the subjects. Both subjects and supervisors were instructed on how to use the exoskeleton. During exoskeleton sessions, which consisted in walking tasks, the users were invited to freely express their opinions on the experience, following semi-structured interviews too. Meanwhile, objective observations (e.g., asymmetries in posture of legs skin irritation in contacts points, user's tendency to self-rotate) were collected to integrate and confirm the insights offered by the subjects' opinions.

According to the reports, the participants especially focused on issues related to comfort, leading to e.g., structural refinements of the braces (as described later in section Braces). They also paid special attention to safety: for example, this made the developers improve the gait patterns via software to approach a more stable trajectory (as explained in section Control System). These evaluation sessions resulted in the spontaneous emergence of comfort and safety as additional requirements, which were set along with those listed in the previous section. Hence, they were added to the preliminary list of five requirements addressed previously and were included in the development process to be taken into consideration for further revisions of the device.

Table 2 presents the full list of requirements set for the development of the Twin exoskeleton.

**Table 2:** Requirements of the TWIN exoskeleton.

**#**	**Requirement**
1	Lightweight and autonomous portability
2	Quick and autonomous donning and setup
3	Stability when standing/walking
4	Cost effectiveness
5	Long battery life (>2 h)
6	Comfort
7	Safety

## Mechatronic Design of the TWIN Lower Limb Exoskeleton

The whole development process was guided by the exploratory and formative processes described in the previous section. It indeed featured a continuous exchange of information between developers and end users (i.e., patients and clinicians) to incrementally and iteratively validate the design choices and the exoskeleton's components (e.g., structure, braces, control) once they were implemented.

The user-centered design approach adopted in this work was not focused on the betterment of exoskeleton performance in the conventional sense: our goal was to develop novel device concepts which could enable new use-case scenarios for the improvement of usability-related aspects of the device.

### Concept Layout Design and Iterative Co-creation Process

**Figure 3:**
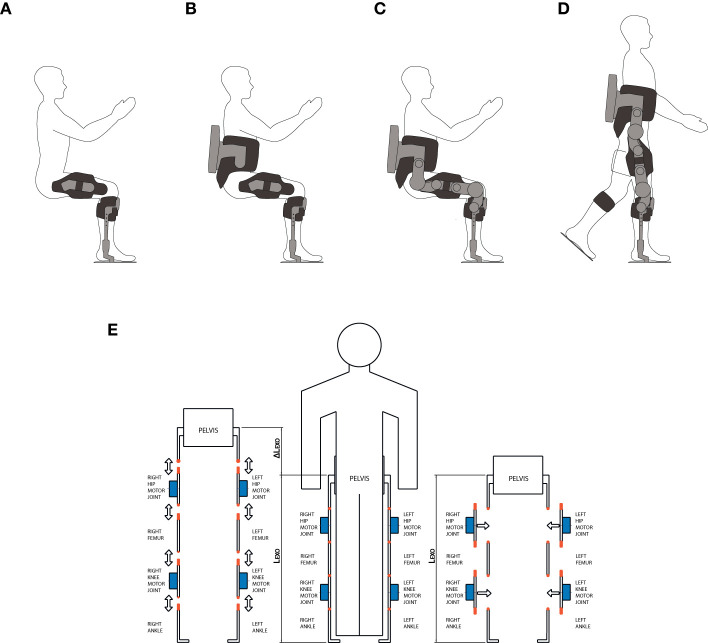
The modularity design concept adopted in the development of the TWIN exoskeleton. **(A,B)** The user can freely wear the lighter and smaller parts of the exoskeleton before, after or between two exoskeleton sessions for ease of transportation and device management. **(C,D)** The user can wear the motor and battery modules as well when he needs to use the exoskeleton. **(E)** The lateral mounting modular design concept. Considering the in-line type of mounting shown in the left, donning requires additional space (Δ_LEXO_), in the limb direction. Because of this reason, unless the exoskeleton is mounted following a specific sequence (either bottom-up or top-down), this scenario may result in awkward donning operations. On the contrary, the adopted lateral mounting system does not require any additional space to be mounted and is free from donning sequence constraints.

Based on the user-centered requirements outlined in Table 2, a concept study was carried out to define possible layouts of the exoskeleton to guide the mechatronic design. Requirements #1, 2 have a few implications: (1) the device must be donned on and off by the subject quickly and autonomously (2) it should be meant for a joint use in combination with the wheelchair, and (3) it should be compatible with personal transportation vehicles (Figure 2B). Notably, the only existing commercial exoskeleton that deals with these issues is the Parker Hannafin's Indego. It indeed presents a modular structure made of five modules (waist, R/L thigh, and R/L shank-foot) which is meant to be autonomously assembled or disassembled by the user and to facilitate transportation and donning operations. The modularity concept has therefore been integrated within this device which effectively represents a step ahead toward usability in the context of personal use: this exoskeleton is therefore considered as the reference gold standard for this work. Inspired by the design concept of the Indego exoskeleton, we developed TWIN by adopting the idea of a modular structure which could be autonomously assembled and by extremizing modularity with the goal of further impacting on user's autonomy. Indeed, the modules which compose the TWIN exoskeleton have been increased to a number of 9. These are: waist, R/L hip motor, R/L thigh link, R/L knee motor, R/L shank link, and foot. We decided to minimize the number of overall actuators (hip and knee motors only) to comply with requirements #1 and #4. One of the advantages of high modularity is that the exoskeleton can be quickly disassembled by the user into small sub-units, so as to facilitate its management and transportation. Indeed, we decided to design a concept layout which allowed to divide the modules into two main groups. The first group includes the heavy, cumbersome and costly parts (i.e., motor modules and batteries), whereas lighter, inexpensive and “tailored” components can be classified into the second group (links and braces). This choice aims to bring direct benefits to both the production cost and usability perspectives and further differentiates our work from the Indego exoskeleton. Thanks to TWIN's design, the patient can wear the modules that less hinder the degree of movement and autonomy, i.e., the links and braces, before, after, or between exoskeleton sessions, Figures 3A–D. This brings a number of usability-related benefits:

1) it minimizes the number of needed donning operations between sessions (only motors and batteries need to be assembled to start a session);
2) it greatly simplifies transportation of the device by allowing the user to carry only the motor and battery modules in a hand luggage;
3) it facilitates compatibility with the wheelchair and personal vehicles.

Furthermore, given the high number of modules, we strategically decided to make the motor units to be mountable laterally to further facilitate donning and assembling operations. From the usability perspective, we argue that this method is to be preferred with respect to “in-line” mounting such as that employed in the Indego exoskeleton. Indeed, the latter forces the user to perform a specific donning sequence and may result awkward as it requires additional axial space to be mounted, as shown schematically in Figure 3E. Instead, our approach facilitates donning and it allows the user to choose the mounting sequence that can best adapt to the specific context, Figures 3A–D. This is another step toward usability, which further differentiates TWIN with respect to the Indego exoskeleton.

From the production cost perspective, the extremization of modularity directly implies saving because the “tailored” parts, i.e., braces and links, which need to be produced in various sizes, are physically separated from the high cost components, i.e., batteries and motors. This facilitates economy of scale in the production of the costly modules and is therefore agreeable with Requirement #4. On the other hand, requirements #3 and #5 were not directly related to the physical layout of the exoskeleton, and were instead implemented through appropriate control, and electrical dimensioning, respectively.

Finally, the two requirements emerged in the formative phase, i.e., #6 and #7 on comfort and safety, have been be considered in all aspects of the design, ranging from e.g., the development of the braces to the implementation of the gait patterns as explained in the following sections.

### System Overview and Architecture

Based on the requirements defined in Table 2, the anthropometry of the European population, direct comparison with other existing exoskeletons, and engineering constraints, TWIN's specifications were broken down and set accordingly with the parameters in Table 3. The target walking speed was not prioritized accordingly with the results shown in Figure 2. Its target weight has been set to be realistically achieved using off the shelf mechatronic components. In fact, as the full weight of lower limb exoskeletons for SCI might result rather high for enabling the user to perform the operations required in autonomous use, we decided that the structure of TWIN should have been “broken down” into a number of modules, each featuring much smaller mass than that of the full device. Furthermore, the weight of the exoskeleton is supported by the structure of the device itself through the soles. Therefore, the user won't bear the weight of the exoskeleton during use. We hence focused on providing an answer to weight issues highlighted by requirement #1 by extremizing the modularity of the device, so as to allow the user to handle small and light modules that could easily be managed individually.

**Table 3:** User-driven specifications of the TWIN exoskeleton.

**Type**	**Value**
Target walking speed	1.5 km/h
Max patient's weight	110 kg
Target weight	20 kg
Battery autonomy (continuous usage)	3 h
Min-max sizes	5^th^ to 95^th^ percentile

**Figure 4:**
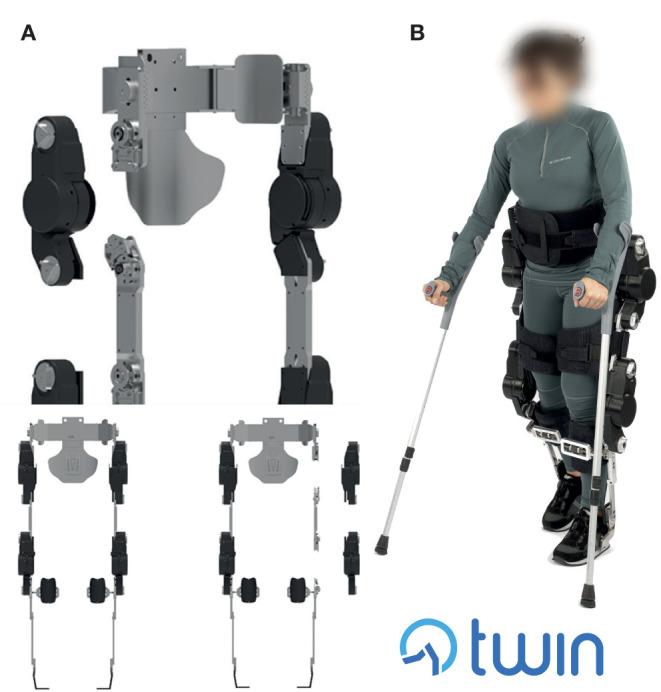
**(A)** TWIN's modular structure and lateral mounting of the modules, **(B)** The TWIN lower limb exoskeleton in walking mode worn by a subject. The person wearing the exoskeleton gave permission for the use of her image.

The four actuation modules can be easily donned on and off by means of lateral quick release connectors placed on both ends of each actuation module, which implement the key concepts explained in the previous section to facilitate the implementation of requirements #1 and #2. The novel quick release system has been custom designed and is a crucial component as it opens up to new use-case scenarios in the field of personal use of lower limb exoskeletons. Still, the design of this subsystem is a major technical challenge that implies critical electromechanical design which must ensure the transmission of both the structural mechanical load and function of electrical connector. Indeed, this component is subject to high values of stress when the exoskeleton is in use, which need to be borne by the mechanical structure, and at the same time it needs to provide continuity in delivering voltage supply and safely stream data throughout the whole structure. Regarding the control of gait parameters, they can be set using a mobile device-based GUI, whereas each step is triggered by means of an Inertial Measurement Unit (IMU)—based system. The actuation guarantees a maximum walking speed of 1.5 km/h on patients weighing up to 110 kg. Structural parts (pelvis, femur, tibia) and braces of different sizes are provided to adapt the device to the anatomy of the patient. The battery pack is located at the back of the device and guarantees up to 3 h of continuous operation. The full device weighs 23 kg and is shown in Figure 4.

Finally, a custom motherboard located in the battery pack area behind the back of the user is employed as central control unit (CCU) to coordinate the actuation units and provide measurements and diagnostics. Particularly, a Xilinx XC7020 Zync-7000 series system on chip (SOC) executes the high-level control.

Regarding the autonomy of the battery, this specification has been set to be sufficiently high, allowing to perform 1–2 walking session with some degree of reserve. Finally, the exoskeleton can be worn and support patients' weight so as to cover the vast majority of the European population, in the range 5^th^-95^th^ percentile.

### Quick Release System

The challenge was to develop a mechanism system with lateral release which could bear the complex, multi axial, force-torque load imposed on the structure, at the same time guaranteeing patient safety, high structural stiffness to comply with requirement #3, power supply and data streaming continuity. Despite the fact that “in-line” coupling layout (e.g., Indego style) might seem preferrable because it copes well with both axial load and bending moments, this choice has again been discarded to prioritize device usability and ease of donning (requirement #2) through lateral mounting. The lateral quick release system was specifically designed to require little manual effort for the patient during use, at the same time ensuring high mechanical and electrical safety, following the IEC 60601 medical electrical devices safety standard (IEC, 2020).

#### Mechanics

**Figure 5:**
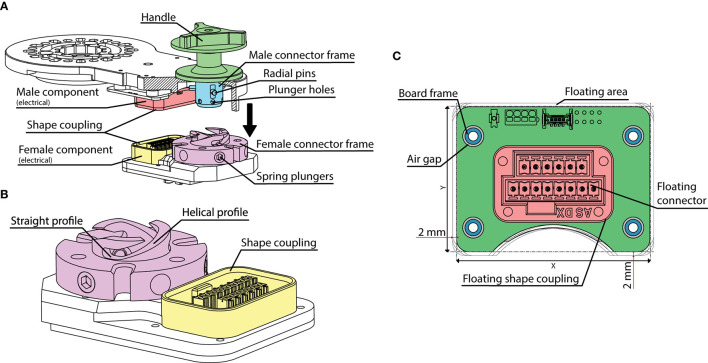
The quick release electromechanical connector. **(A)** General assembly and main components of the mechanism. **(B)** Detail of the female component. **(C)** The floating electrical connector board. The floating connector and its frame (Floating shape coupling) are fixed to the board. An air gap is left between the board's fixation holes to implement a floating area of 2 mm in both the vertical and horizontal directions.

The mechanism is shown in Figures 5A,B: the male component is composed of a large pin that features three further small radial pins equally spaced by a 120° angle, whereas the female counterpart is made by a hollow cylindrical shaped part that was machined to present three helical-shaped grooves. When the male part enters its counterpart, the three radial pins get engaged into the three corresponding grooves machined on the female component by means of manual application of torque on the handle in the clockwise direction. Given the helical shape of the grooves in the female part, when the user applies torque in the clockwise direction, the male part of this system, which is located to each extremity of any of the motor modules, slides along its axis until the mechanical end-stop is reached, when the pin reaches the end of the groove machined on the female part, Figure 5B. The groove profile, in its final part, transitions from helical to straight, to ensure the stability of the male part when engaged within the female, Figure 5B. Furthermore, three spring plungers have been radially arranged on the female component.

These get engaged with three corresponding holes machined on the main pin of the male component when the handle reaches its end stop, with the dual function of providing acoustic feedback to the user on the successful locking of the system and to additionally ensure mechanical locking safety, Figure 5. Indeed, when the plungers are engaged, they lock the male part in its end stop position to avoid accidental disengagement. Hence, to unlock this part, it is necessary to pass a certain torque threshold value which can be regulated by appropriate preload of the plungers' springs which was set to 7 Nm. Please note that, although the torques applied to the structure during exoskeleton use are significantly higher than the required level to unlock the quick disconnect system, the shape coupling profiles of this component, Figure 5, decouple the structural loading from the torque loading of the handle. As a result, the male and female components are stressed in traction/compression mode. The main male pin and its counterpart are both machined using 41CrAlMo7 hardened steel, while additional gas nitriding treatment has been performed on the surfaces. This allows to increase Vickers hardness to a value of HV1150, which guarantees very high wear-resistance performances and reduced friction. FEM simulation was also performed on the main components of this critical system to validate their design. The load scenario used in this simulation replicated that measured by a motorized dummy exoskeleton which was equipped with force/torque sensors (please refer to section Structure and Actuation Unit for more details on this device). To obtain this data, a healthy subject simulating leg impairment wore the exoskeleton while position controlled to perform the walking patterns described in our previous work (Vassallo et al., 2020). The worst-case load scenario is summarized in Table 4, where the Y axis represents longitudinal axis of the lower limb, the Z axis is the medio-lateral axis and the X axis completes the triad.

**Table 4:** Load configuration used for the FEM simulation.

**Load**	**X**	**Y**	**Z**	**Total**
*Force (N)*	250	−300	290	486
*Torque (Nm)*	56	19	−45	74

Results from this simulation confirm that the critical components, i.e., the main male pin and its counterpart, are able to withstand the load, reaching a peak stress of 441 MPa, which allows validation of the design, taking into account a safety factor of about 2. The male quick disconnect halves are placed on both ends of the actuation modules, whereas their receptacles are placed at the ends of the structural parts described in section Structure and Actuation Unit.

#### Electrical Connections

Given that requirement #2 expressed the need of performing quick donning and setup, we decided to develop an embedded quick release that could incorporate electrical connections (voltage supply and CAN communication) as well, so as to allow to achieve both mechanical and electrical connection in one simple step. This solution additionally favors usability by eliminating any external cabling which could possibly result in entanglements with external objects during use. From the technological perspective, though, this constitutes a further challenge. Indeed, ensuring stable electrical continuity over a connector that is subject to considerable mechanical stress is particularly challenging, especially in this case where compactness is paramount and where the applied load is a combination of multi-axial forces and torques. To solve this issue, we designed a system which mechanically decouples the electrical connection from the structural parts of the connector. Indeed, the female electrical connector was mounted on a “floating board,” which is able to freely move on its plane up to 2 mm on both horizontal and vertical directions. This range of movement is so small that it does not pose robustness problems if all the cable routing and soldering of the terminations are made appropriately. This was achieved by means of a specific loose fit between the floating board and the structural frame, Figure 5C. This prevents unwanted stress to be generated by deformation of the mechanical structure when under stress. The centring between male and female electrical connectors is guaranteed by the custom-made connector frames which allowed shape coupling as shown in Figure 5. The electromechanical design choices adopted in the development of the quick release system, have permitted to obtain the highest safety of this critical component, which additionally allowed TWIN to obtain compliance to IEC 60601 standards.

### Actuator Sizing

**Figure 6:**
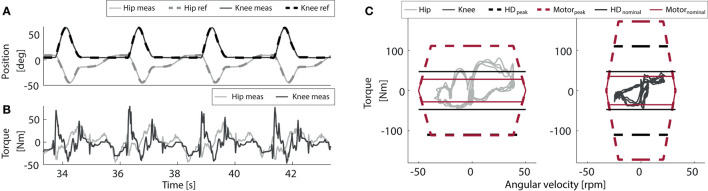
Torque-velocity characteristic of the actuator modules of the hip and the knee joints, transposed for a 110 kg patient weight. **(A)** Trajectory references and tracked positions. **(B)** Measured torques. **(C)** Torque/speed plots and mechatronic limits of the actuation system.

The dimensioning of the actuators and battery unit of the TWIN exoskeleton was based on the specifications set in Table 3. Given that the walking pattern of exoskeletons for this type of application is significantly different with respect to healthy subjects' physiological gait velocity/torque profiles, we decided not to use human biomechanical gait data as reference values for the design as they would result unrealistic and require significantly higher mechanical power than needed. Indeed, motion patterns replicated by exoskeletons are rather different compared to those of healthy subjects. To obtain more realistic data, we therefore developed and used a custom-made “dummy” motorized lower limb exoskeleton that was fully sensorized for the purpose of recording the load applied to the exoskeleton actuators and structure during position-controlled walking, standing and sitting and use them for appropriate actuators dimensioning. A classical robotic trajectory was set as reference for the position-controlled system, Figure 6A (T1 as explained in Vassallo et al., 2020), and a healthy male subject, 60 kg, wore the exoskeleton performing a few steps simulating full leg impairment.

The recorded joint-space velocity-torque profiles of the knee and the hip, Figures 6B,C, were then scaled up to respect the patient's weight and walking speed specs set in Table 3, so as to properly size the motor-gearbox modules accordingly to worst-case conditions. This data was later processed by a dynamic model of the actuator module to determine an appropriate combination of motor and gearbox, which could respect the required velocity, torque and power values. A torque-velocity graph that reports the results of these simulations is shown in Figure 6C. All the results in Figure 6 show that an appropriate combination to be used for the actuation of the hip and knee is composed of the brushless motor Maxon EC90 and the Harmonic Drive CSD-25-100-2A-GR-BB 100:1 gearbox. The motors employed for the knee and hip had different nominal voltages, 24 and 36 V, respectively, to meet the different torque/speed requirements of these two joints. Each motor is controlled by a custom-made board which features a PWM controlled three phase Mosfet bridge inverter, which compensates the variable input voltage with the motor control algorithm.

### Battery Units and Electronic Architecture

Regarding the battery unit, referring to requirement #5 and the specified target value set in Table 3, we assumed an exoskeleton's battery life of 3 h. Assuming the device operating in typical conditions, i.e., walking at the set target speed as defined in Table 3 performed by an average european weight wearer (70.8 kg, Walpole et al., 2012), we computed the average power consumption required by the TWIN Exoskeleton to compute the task, which was about 59 W. Hence, it results that battery pack charge should be at least 177 Wh. This value, combined to a nominal voltage that is compatible with the chosen motors and a peak power output that can deal with the worst case operative conditions (subject weighing 110 kg walking at 1.5 km/h), defined the main target values for the battery unit. We therefore designed a battery unit made by a combination of two Accutronics CMX820P Li-ion battery packs, which can deliver a total energy of 189.4 Wh and a peak power output of 482 W, with a nominal voltage of 28.8 V and maximum current peak of 15 A, which are compatible with our requirements. The batteries are mounted in a docking system that allows to change batteries when needed to simplify cabling and ease of replacement. Each pack is monitored by an internal BMS which to the motherboard via SMBus. The BMS includes safety, diagnostics, and communication functionalities. The two battery packs are paralleled by an or-ing ideal diode circuitry, and the current delivered to each leg is measured via shunt current monitors.” Furthermore, these battery units are IEC62133 certified and can therefore assure safety of the TWIN exoskeleton accordingly with the IEC60601-1 norms.

The electronics architecture of the TWIN exoskeleton is presented in the Supplementary Figure 1. The main component of this system is the custom main motherboard (named SMEX in the diagram) which is based on a Xilinx Zynq-7000 SOC running a Linux OS. The SOC interfaces to a variety of sensors; including two independent IMUs, doubled for redundancy and cross checking, a battery voltage sensor and two separate leg current sensors, used for monitoring, logging, and diagnostic purposes. The custom SMEX motherboard also implements several communication peripherals, including Ethernet, Wi-Fi, and Bluetooth for diagnostics and interface to host devices (PC or tablet), two separate CAN Bus lines (one for each leg) for internal communication to the motor boards, and one SMBus line for the communication to the battery packs. The SMEX motherboard exchanges CAN bus packets with the motor control boards. Particularly, the SMEX sends to the motor control boards the reference set-points, according to the selected control strategy, while the motor control boards sends messages back regarding joint absolute position, status, and motor current readings. Each active joint features a custom motor control board, including a PWM controlled three phase Mosfet bridge inverter, to drive BLDC or PMSM motors from 18 to 60 VDC, up to 35 A motor current. The motor current is monitored via low-side shunt current monitors.

Regarding the available sensing, each joint contains a fast-shaft quadrature encoder (6,400 pulses per revolution), used for motion control, three phase hall-sensors used for commutation, and a custom slow shaft absolute potentiometer, that is used for calibration purposes and redundancy. The reference joint trajectories are treated as setpoints that are tracked by using a PI controller.

### Structure and Actuation Unit

**Figure 7:**
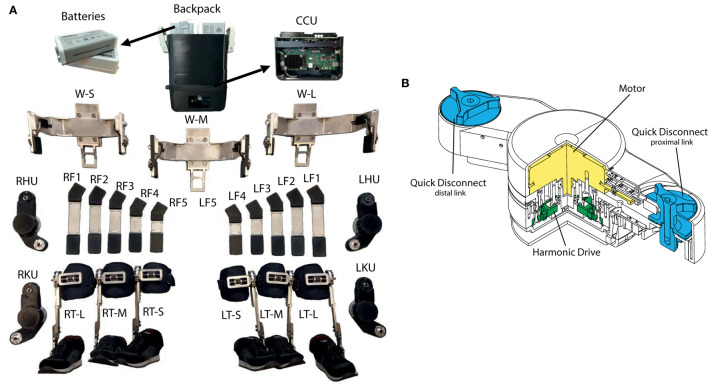
**(A)** The main structural parts of TWIN. From the top, batteries, and backpack, Central Control Unit (CCU); W-S, W-M, and W-L are three waists sizes (small, medium, large); RHU/LHU are right and left hip motor unit; RF1,…, RF5 and LF1,…, LF5 are right and left femur sizes; RKU/LKU are right and left knee motor unit; RT-S, RT-M, RT-L, LT-S, LT-M, LT-L are right and left tibia sizes; AFOs are located inside the shoes, ranging from 38 to 46 EU shoe size. **(B)** The actuation module used for actuating the exoskeleton. A Maxon EC90 flat motor is coupled to a 100:1 CSD Harmonic Drive gear. Two quick disconnect systems are located at both proximal and distal link extremities.

The structure of the TWIN exoskeleton is largely made by welded Al7075 T6 aluminum alloy profiles. This choice was made to keep the overall structural weight low as well as to minimize costs as outlined by requirement #4. Four main structural parts can be identified. These are: (1) waist; (2) femur; (3) tibia; (4) foot, Figure 7A. The waist was designed so as to replicate a “C-shape” profile which accommodates the proportions outlined in Dreyfuss (1993). Three sizes of waist have been designed to cover the set anthropometry requirements (Dreyfuss, 1993). This part is also responsible for housing the battery pack and CCU, that are located at the back of the device in separate modules that can be disconnected separately thanks to a custom-made dock that is rigidly fixed to the waist module. This allows ease of battery replacement. The femur modules are located between the hip and the knee motors and are composed of a straight link that employs a rectangular shaped Al7075 T6 aluminum alloy profile which ends on both sides with receptacles of the quick disconnect system. A total of six sizes of this link have been manufactured to accommodate the anatomical variation among the population with sufficient precision. Indeed, given that the gap between one size and the next is 2 cm, a maximum misalignment of 1 cm can manifest between the motor and the physiological joint^1^.

The tibia comes in one size that is able to fit the different patient lengths thanks to a regulation system that was designed for the purpose. The upper end mounts a quick disconnect receptacle that is employed to connect this segment to the knee motor module. The lower end of this segment connects to the foot.

The foot comprises a passive elastic ankle joint, which is then connected on its lower end to a custom carbon fiber-based footsole. Passive elasticity is implemented using a simple mechanism made of linear compression springs placed antagonistically which can be preloaded by means of two socket screws. These screws can also be employed to set the ankle joint's equilibrium point, as well as its stroke, to accommodate its configuration to each patient's need, Figure 7A. The ankle mechanism design was inspired by that implemented in the ReWalk exoskeleton.

The structure has been dimensioned using the force/torque data recorded in the dummy exoskeleton in the experiments explained in section Actuator Sizing, employing a safety factor of 2.5. Finally, these calculations have been validated by means of FEM simulations.

Regarding the actuation unit, the Maxon EC90—Harmonic Drive CSD-25-100-2A-GR-BB 100:1 gearbox assembly is implemented as shown in Figure 7B. The proximal link is connected to the motor's frame whereas the distal link is connected to the Harmonic drive's output shaft.

### Braces

**Figure 8:**
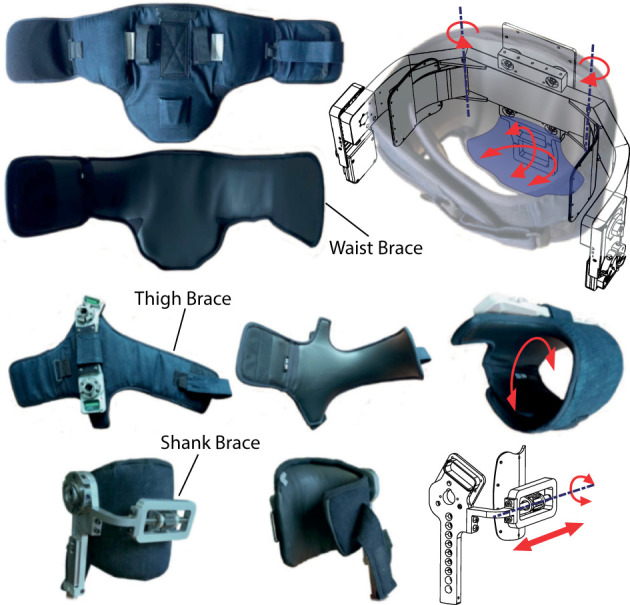
The brace modules—The waist (top), femur (middle), and tibial (bottom) braces and their DOFs.

The interface between the patient and the machine stands as a critical aspect of exoskeleton development. Hence, they should converge on a variety of aspects such as: ergonomics, comfort, safety, anthropometry, and aesthetics while maintaining biomechanical and functional requirements. Although comfort did not emerge directly from the initial user need analysis phase, we implicitly took this aspect as priority during the formative phase as highlighted in section Formative Phase: Iterative Improvements of the Device where we discuss about the role of their contribution within the formative assessment of usability. Indeed, the delicate skin of SCI patients can be easily damaged by the generation of unwanted forces or pressures on the contact points. This holds true also if the user wears the braces over their own clothes as the avoidance of these unwanted effects depends on a combination of good structural design (mostly to avoid pressure concentrations in the orthogonal direction) as well as on a good choice of materials to avoid chafing effects of the brace or cloth against the user's skin. Hence, the braces must guarantee a safe and comfortable interfacing with the wearer (Requirements #6 and #7, Table 2) so as to in turn accommodate safe and stable walk as outlined by requirement #3. TWIN is currently equipped with a pair of thigh and shank braces for each leg and a waist brace as the uppermost connection to the patient, for a total of five human-machine interfaces Figure 8.

All braces employ Velcro^®^-based straps to achieve correct tightening during donning procedure. The inner region in contact with the patient is made of spandex material, which ensures biocompatibility and reduces shear forces on patient skin, main causes of skin lesions. The outer region is made using denim for durability. The waist brace is composed of a central portion which is directly connected to the structure by means of bolts and two lateral bands wrapped around the patient and can be tightened on the front. This brace additionally hosts a pair of stiff wings that are hinged on the waist structure and can rotate on the transverse plane: this additional DOF helps to accommodate lateral weight-shifting on the support leg during gait, while providing maximum patient-exoskeleton connection of tilting movements in the sagittal plane, achieving higher controllability of the machine's step trigger (please see section Control System). Finally, this brace houses a coccyx support connected to the waist structure, which avoids excessive lumbar hyper-lordotic postures. A semirigid shell, which can tilt in the transversal and sagittal plane, is placed on top of the coccyx support inside the brace to allow rotations of the sacrococcygeal region to avoid generation of shear stress on the patient's skin (Figure 8—top right). The frontal portion of the brace acts as a thoracic stabilizer preventing the patient from collapsing, which is critical for SCI patients with higher level of injury. Each thigh can partially rotate (see Figure 8—middle right) around the femur, which ease donning/doffing procedures. The shank brace is composed of a hinged semirigid plate which hosts the patient tibia and can tilt in the sagittal plane. The medio-lateral position of the brace can be adjusted to account for different postures or deformities in the knee joint by means of a leadscrew mechanism (see Figure 8—bottom right). This mechanism is inspired by that employed in the ReWalk exoskeleton shank brace design.

## Control System

As the patients targeted in this work are complete SCI, we opted for a position control-based scheme to provide full support to the patients during use. The identification of predefined gait trajectories to be employed on lower limb exoskeletons is typically obtained by fitting mathematical curves to temporal sequences of desired joint angles that are often inspired by biological gait patterns. In contrast to this, in Vassallo et al. (2020) we proposed predefined gait trajectories for the TWIN lower-limb exoskeleton, generated in Cartesian space, by using a basis function interpolation method which was designed so as to maximize stable walk as outlined by requirement #3. Such approach allows to guarantee the length and clearance of each step, despite the variation of tibia and femur lengths. Thanks to this, the trajectories are fully parametrizable to better fit user needs. In any of the gait patterns, steps are triggered by reaching a set of two torso inclination thresholds. Torso inclination angles were measured by the IMU sensor located in backpack of the device.

### Computing the Reference Trajectory

**Figure 9:**
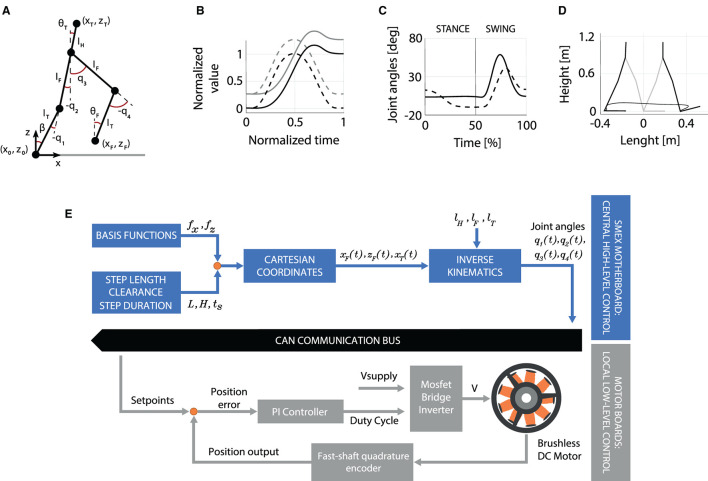
**(A)** The kinematic model of TWIN. **(B)** The basis functions *f*_*x*_ and *f*_*z*_ are represented by the continuous and dashed lines, respectively. **(C)** Reference joint trajectories, in position control, during a stride. **(D)** Cartesian trajectory of the swing foot during a step. In **(C)** and **(D)** plots, the step is ~0.8 m long with a peak height of ~0.20 m. **(E)** Conceptual block scheme showing the control architecture of the TWIN lower-exoskeleton.

The kinematic model of the TWIN exoskeleton is shown in Figure 9A. It has been developed based on Denavit-Hartenberg convention, considering the bilateral actuations of the knee and hip as *q*_1_, *q*_3_ and *q*_2_, *q*_4_, respectively. Conversely, the tibia and femur links length, and hip-COM distance, are defined as *l*_*T*_, *l*_*F*_, and *l*_*H*_. These lengths are fixed and do not change during the whole session with a patient. We define θ_*T*_ as the tilt angle of the torso with respect to the frontal plane, β the flexion angle of the support ankle, and θ_*F*_ the orientation of the swing ankle. Given the absence of sensors on the ankle joints, β cannot be measured. Therefore, we estimate its value, that is comprised between two values β_min_ and β_max_, which are set according to the patient's need during the setup phase by using the regulation screws as explained in Section Structure and Actuation Unit.

### The Design of the Gait Pattern

The gait pattern is generated by an interpolation approach, consisting in multiplying basis functions, normalized in amplitude and over time, and depend on the desired step length L and height H. The reference trajectories are represented in the Cartesian space by the following Equations: (1) $x_{F}\left(t\right)=x_{F}^{0}+L\;f_{x}\left(t\right)$, (2) $z_{F}\left(t\right)=z_{F}^{0}+H\;f_{z}\left(t\right)$, (3) $x_{T}^{0}+L\;g_{x}\left(t\right)$, where (*x*_*F*_, *z*_*F*_) represent the swing foot coordinates, and *x*_*T*_ the torso ones. $x_{F}^{0}$, $z_{F}^{0}$, $x_{T}^{0}$ represent the foot coordinates at the beginning of the step. The basis functions *f*_*x*_, *f*_*z*_ define the walking shape while *g*_*x*_ is a 6^th^ order polynomial. These functions are normalized and assume a value which can range from 0 to 1. A more detailed description of the definition of the basis function is given in Vassallo et al. (2020). Given the reference trajectory in the Cartesian space *x*_*F*_, *z*_*F*_, *x*_*T*_, we compute the joint angles based on the following assumptions: (i) θ_*T*_ = β + *q*1 + *q*2 with {β ∈ ℝ, β_min_ ≤ β ≤ β_*flex*_}, (ii) θ_*T*_(*t*) = 0, ∀*t* ∈ [0, *t*_*S*_] where *t*_*S*_ is the step duration.


(1) $$\begin{array}{r} q_{1}=\sin^{-1}\left(\frac{x_{T}-\left(l_{T}\sin\left(\beta \right)+l_{H}\sin\left(\theta_{T}\right)\right)}{l_{F}}\right)-\beta \end{array}$$

(2) $$\begin{array}{r} q_{2}=\theta_{T}-q_{1}-\beta \end{array}$$

(3) $$\begin{array}{r} q_{3}=\cos^{-1}\left(\frac{a_{x}^{2}+a_{z}^{2}-l_{F}^{2}-l_{T}^{2}}{2l_{F}l_{T}}\right) \end{array}$$

(4) $$\begin{array}{r} q_{4}=\tan^{-1}\left(\frac{z_{F}-z_{T}}{x_{F}-x_{T}}\right)-\tan^{-1}\left(\frac{l_{T}\sin\left(q_{3}\right)}{l_{F}+l_{T}\cos\left(q_{3}\right)}\right) \end{array}$$

A representation of the gait pattern is shown in Figures 9B,D. Particularly, Figure 9B shows the basis functions, that are employed to compute the joint angle trajectories shown in Figure 9C. Figure 9D shows the corresponding cartesian representation of the foot trajectory on the sagittal plane.

An overview of the described control system is shown in Figure 9E: the basis function *f*_*x*_, *f*_*z*_, the desired step length *L*, the clearance *H*, and the step duration *t*_*S*_ are inputted to the high-level control, which in turn extrapolates time-continuous reference trajectories in the Cartesian space. Then, the inverse kinematics equations of the system allow to compute the related joint angles *q*_1_(*t*), *q*_2_(*t*), *q*_3_(*t*), *q*_4_(*t*) based on the specific exoskeleton dimensions *l*_*H*_, *l*_*F*_, *l*_*T*_. Finally, the reference joint positions are sent to the four motors via the CAN-bus. At the motor-board level, the local low-level control transforms these inputs into a PWM's duty-cycle value, which is returned by the PI controller, to drive the motor.

The development of the gait pattern was made to comply the following aspects: (1) stability and safety, which was priority according to requirement #3 and requirement #7, (2) similarity with those employed in existing exoskeletons, (3) experience gained in pilot trials. The resulting trajectory is characterized by a marked clearance and an emphasized heel-strike to comply with requirement #3. In fact, in humans, the heel-strike to toe-off movement has an important role, widely studied in literature: the foot arches compliance reduces metabolic energy consumption during locomotion, help balance and, consequently, improve stability (Stearne et al., 2016). Most importantly, this approach is also beneficial to the safety of the patient because it is meant to avoid the potential hazard caused by accidental stumbling (requirements #3 and #7).

### Trigger

**Figure 10:**
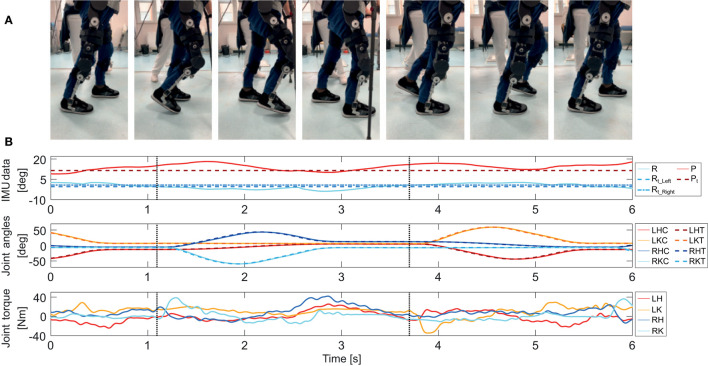
**(A)** Walking sequence of the SCI subject performing straight walk in controlled environment. **(B)** (Top) Roll and pitch angles as recorded from the IMU, and corresponding thresholds, (Middle) reference and recorded angular joint positions, (Bottom) torques delivered by the joints. The dotted vertical lines denote the step trigger. A pause of 0.15 s between the trigger detection and the actuation of the motors was intently set to signal the patient a step was about to be performed.

The trigger scheme's function is to initiate steps based on the intention of the user by elaborating signals coming from the IMU sensor located in the CCU compartment. The pitch P and roll R angles are defined as the tilt of the waist unit with respect to the frontal and sagittal planes, respectively. When these angles both pass the threshold values P_t_ and R_t_Left_, or R_t_Right_, the step trigger is activated. These parameters can be set according to patient needs. This functionality can be observed in the plots shown in Figure 10B.

## Mechatronics System Evaluation

The validation of the device's mechatronics as well as a preliminary evaluation of the ergonomics and the feasibility of the designed gait trajectories have been carried out by testing the device on healthy subjects and on one SCI subject, as discussed in section Formative Phase: Iterative Improvements of the Device as part of a formative assessment of usability which focused, in this case, on the efficacy and the efficiency of the advanced prototype of TWIN which is the result of the first 36 months of development and formative phase. The subjects were asked to perform straight walk from a starting to a final point located 10 m away at their preferred speed. Figure 10A reports a sequence of images of the patient, while the plots of IMU data, position, torque, and of an extract of two steps are reported in Figure 10B. This task was freely repeated by the subject in each session—after 30 min the trials were stopped in order to monitor the conditions of the participant (especially on potentially sore areas—coccyx and tibia). The joints' torque, speed, as well as the IMU data were recorded during trials with the patient and were used to assess and validate the mechatronic design. Figure 10B shows the trend of lateral and frontal inclination of the exoskeleton, as well as the angular positions and torques of the joints, while the step trigger times are denoted by the vertical dotted lines.

## Discussion

Thorough effort was dedicated during the whole development phase to satisfy the user-centered requirements set in the initial field research reported in Table 2 (system portability, donning and setting autonomy, standing/walking stability, cost effectiveness, long battery life) and in the formative process (comfort, safety). These requirements involved significant implications to the mechatronics of the device according to a strict adoption of quality management system for medical devices (i.e., ISO 13485:2016), and in function of its usability, as defined by IEC 62366-1:2015. Consequently, the safety of the device (Requirement #7) was tested according to the IEC 60601 safety standards, while other usability-related aspects, like comfort, were assessed by means of a periodical analysis of feedback from both expert clinicians and users. Furthermore, a preliminary clinical evaluation was conducted on one SCI subject for a first field test of the whole system, while TWIN's distinctive features with respect to both commercial and research exoskeletons have been considered in the evaluation. These investigations enriched the formative assessment itself by exploiting user research to iteratively improve the different prototype versions of the TWIN system as required by the standards of IEC for the usability engineering of medical devices.

Regarding comfort, i.e., Requirement #6, the SCI user verbally expressed high appreciation of our bracing system compared to the exoskeleton he daily uses at home, i.e., ReWalk. The user also did not report any skin injuries in the points of contact with the exoskeleton and no signs of lesion in the critically stressed areas (tibial tuberosity and sacrococcygeal). Similar positive feedback was also given by clinicians, which confirmed its suitability to operate on the delicate skin of SCI subjects. Of course, this cannot be considered as definitive validation but is in fact taken as an initial positive result to feed further iterations of the formative assessment.

Another significant outcome of the clinical test on the SCI subject is that TWIN's trajectory patterns facilitate the stability of the user due to its emphasized heel strike, as explained in detail in our previous work (Vassallo et al., 2020) and this is agreeable with respect to usability too.

Finally, from the mechatronic perspective, plots from the clinical trial on the SCI patient (Figure 10) demonstrate that the system can track the trajectory imposed by the CCU for straight walking at 0.35 m/s. At the same time, the delivered torques are below the nominal torque of the Harmonic drive and well below the peak torque value of 110 Nm. Furthermore, the values of delivered current are within the continuous operation limits of the motors and do not generate heat during functioning, meaning that the actuators operate in their nominal range. This safe operating condition, which is well-below the mechatronic operational limits of the exoskeleton, occurs because the device was operating under the following conditions: (1) the user was an expert exoskeleton user and knew how to use the machine with confidence, (2) the walking speed was about one third of the maximum allowable speed, (3) the task was performed by a user weighing 63% of the maximum allowable weight. Although further trials will be needed to validate this design, e.g., on patients with higher weight, and at higher walking speed, the obtained results are a clear indication that the mechatronic design is reasonably sized.

Furthermore, comparing TWIN with the state of the art, and focusing specifically on the requirements initially outlined in Table 2, we can state that Indego is the exoskeleton which best managed to address Requirements #1 and #2 (Lightweight and autonomous portability, Quick and autonomous donning, respectively), among existing commercial and research devices. In order to improve these characteristics, TWIN introduced a novel design primarily addressed to maximize autonomous use by developing a structure characterized by a higher number of modules with respect to the Indego exoskeleton, coupled with an unconventional lateral mounting solution. These features can greatly facilitate donning and allow the user to mount the modules according to the sequence that can best adapt to the context. In addition, although we slightly exceeded the target weight (TWIN weighs 23 kg), high modularity allows users to keep the lightest (9.8 kg, i.e., 43% of the total weight) and most comfortable modules donned on when the exoskeleton is not in use, accordingly with the considerations made in sections Concept Layout Design and Iterative Co-Creation Process. We hence argue that this solution considerably simplifies autonomy of use and transportation, with the goal of facilitating independent domestic usage that is paramount to guarantee high frequency of use and therefore maximize the benefit provided by the exoskeleton training.

Regarding other requirements, most commercial exoskeletons, such as the Ekso, ReWalk, Indego, to name a few, present battery life that is lower than the target imposed by Requirement #5. Hence, TWIN also offers advantages in terms of battery life, allowing the user to make longer exoskeleton sessions, hence facilitating intensive training.

Regarding Requirement #3, all the cited exoskeletons, to the best of our knowledge, can guarantee stability when standing, whereas a dedicated comparative study would be needed to draft conclusions on walking stability. Nevertheless, we argue that, compared to the “traditional” patterns adopted by e.g., ReWalk or TWIICE, the trajectory designed for TWIN may bring additional benefits related to stability because of its emphasized heel-strike which also facilitates stumble-free walking patterns (Vassallo et al., 2020).

Furthermore, considering all existing exoskeletons, there is still room for improvement on requirement #4, i.e., cost effectiveness. Indeed, although exoskeleton companies are striving to keep low costs, the current market price of these devices make them hardly affordable to the average user. Regarding TWIN, the strategic choice to adopt standard and low-cost components in the device design will allow to set a highly competitive target price in TWIN, which will be close to that of the most inexpensive commercial lower limb exoskeletons.

To provide a general overview of the differences of TWIN's design concept and related priorities we can state that other designs might endow a stable standing/walking, cost effectiveness, long battery life, high comfort, high safety, (i.e., req. #3–7). However, most of these priorities are not jointly considered in a “holistic” way and, in most cases, are not meant to directly tackle autonomous portability and, related to this, quick and autonomous donning and setup (req. #1, 2). An exception can be made for the Indego exoskeleton which adopted a similar modular design strategy to that of TWIN, as explained in section Concept Layout Design and Iterative Co-Creation Process.

Finally, we argue that the employed user-centered approach very much fits the development of healthcare robots such as exoskeletons. Indeed, we experienced great effectiveness to plan and continuously adapt design goals and activities toward the satisfaction of user needs. In addition to this, the employed approach has demonstrated to provide a vast number of insights that drove technological as well as design choices.

## Conclusions

This work presented the design of TWIN, a novel lower limb exoskeleton for personal use of SCI subjects, and the user-centered design approach adopted for its development. This device is the result of a joint effort coming from a tight cooperation between engineers, industrial designers, physical therapists, physiatrists, and SCI patients, which jointly cooperated in an iterative development process, which started with the definition of a set of five user-centered design principles, that were subsequently integrated by two other requirements that emerged during the formative processes. An initial concept layout analysis was presented to show how this device was conceived to maximize usability. The consequent advantages and novelty of the proposed solution, which is mainly based on high modularity and lateral mounting, were highlighted, especially considering the state of the art. A series of iterative tests were implemented as part of the formative evaluation of TWIN, following the requirements established worldwide by IEC 62366-1:2015 for the usability engineering of medical devices. Moreover, preliminary results showed that the device mechatronics is capable of delivering the torque/speed profiles required for a typical exoskeleton session. Overall, the device was assessed positively by the SCI subject and expert clinicians, from both the comfort/ergonomics perspective and feasibility of the walking pattern. From this initial assessment and discussions with users and experts in the field, we claim that the successful design of personal aids must rely on detailed analyses of the needs and the lifestyle of users. Indeed, user-centered design techniques require the implementation of careful analyses of users' need before the design of any prototype. Similarly, the formative assessment needs to be executed since the very beginning and throughout the whole development process. We believe this can only be achieved by means of a rigorous application of user-centered design and co-development approaches, as presented in this work.

Future work on TWIN will include the summative assessment of the device and its clinical evaluation on a larger subject population. In this future study, specific focus will be devoted to the quantitative evaluation of usability.

## Data Availability Statement

The original contributions presented in the study are included in the article/Supplementary Material, further inquiries can be directed to the corresponding author/s.

## Ethics Statement

The studies involving human participants were reviewed and approved by Comitato Etico Interaziendale Bologna-Imola of the Pharmaceutical Department U.O.C. Farmacia Ospedale Maggiore, Bologna, Italy. The patients/participants provided their written informed consent to participate in this study. Written informed consent was obtained from the individual(s) for the publication of any potentially identifiable images or data included in this article.

## Author Contributions

ML and JS conceived the study. ML wrote the first draft of this manuscript. SD'A, CV, CP, MC, SD, MD, AS, SC, GC, SS, LC, NB, CM, JS, EGu, GB, MS, ST, SM, FM, RS, EGr, and LD wrote sections of this manuscript. All authors contributed to the revision of this manuscript, and they read and approved its submitted version.

## Funding

This work was funded by the Istituto Nazionale per la Assicurazione contro gli Infortuni sul Lavoro (INAIL) under grant agreements POR-1 and POR-AI 1/1.

## Conflict of Interest

The authors declare that the research was conducted in the absence of any commercial or financial relationships that could be construed as a potential conflict of interest.

## Publisher's Note

All claims expressed in this article are solely those of the authors and do not necessarily represent those of their affiliated organizations, or those of the publisher, the editors and the reviewers. Any product that may be evaluated in this article, or claim that may be made by its manufacturer, is not guaranteed or endorsed by the publisher.

## Footnotes

^1^This of course holds true for measurements of the user limb lengths that are performed correctly by the clinician/physiotherapist.
