# Supplementary material for: User-Centered Design and Development of the Modular TWIN Lower Limb Exoskeleton
Source: Front Neurorobot. 2021 Oct 7;15:709731. doi: 10.3389/fnbot.2021.709731 (PMC8529015; doi:10.3389/fnbot.2021.709731)
Supplement: Supplementary file 2 [file Image_1.pdf]

Supplementary Figure 1

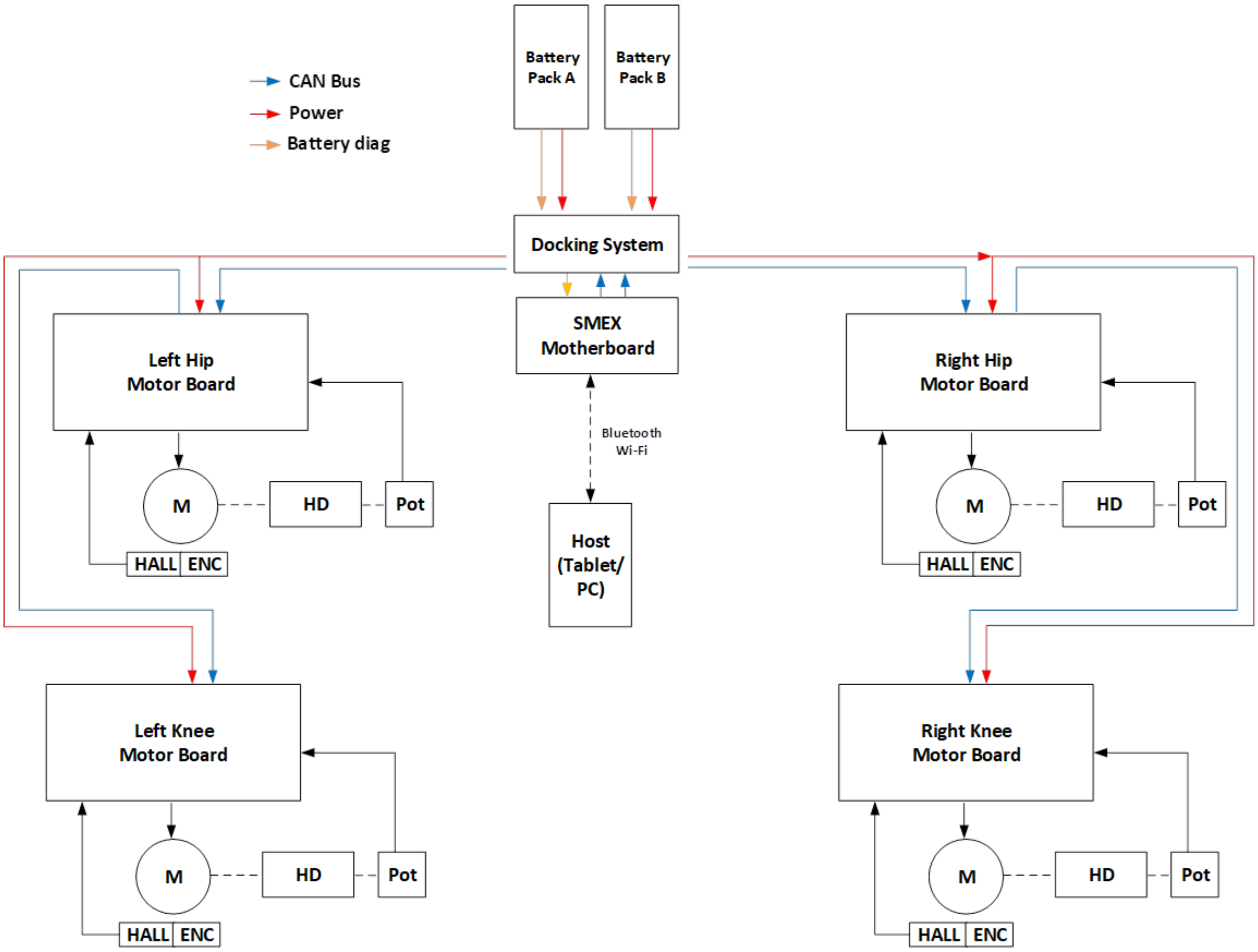

Fig. S1 – The electronic architecture of the TWIN lower limb exoskeleton. The Docking System connects the two certified Battery Packs (A and B) and the SMEX custom motherboard to the exoskeleton motors (marked as M in the figure). The SMEX motherboard controls and monitors the overall system, interfacing to an external host for the tasks configuration and diagnostics. The Docking System easy the routing of power and CAN Bus to the active joints. Each active joint includes a BLDC motor (M) and a harmonic gearbox (HD), controlled by a custom motor control board interfacing to the motion sensors. These are: hall sensors (HALL), incremental fast shaft encoder (ENC) and an analog slow shaft potentiometer (Pot).
